# Interactive neonatal gastrointestinal magnetic resonance imaging using fruit juice as an oral contrast media

**DOI:** 10.1186/1471-2342-14-33

**Published:** 2014-09-22

**Authors:** Owen J Arthurs, Martin J Graves, Andrea D Edwards, Ilse Joubert, Pat AK Set, David J Lomas

**Affiliations:** 1 Department of Radiology, Cambridge University Hospitals NHS Foundation Trust, Cambridge CB2 0QQ, UK; 2 Department of Radiology, Great Ormond Street Hospital for Children NHS Foundation Trust, London, UK; 3 UCL Institute of Child Health, London, UK

**Keywords:** Neonatal, MRI, Bowel, Fruit juice

## Abstract

**Background:**

The objective was to evaluate the use of fruit juice with an interactive inversion recovery (IR) MR pulse sequence to visualise the gastrointestinal tract.

**Methods:**

We investigated the relaxation properties of 12 different natural fruit juices in vitro, to identify which could be used as oral contrast. We then describe our initial experience using an interactive MR pulse sequence to allow optimal visualisation after administering pineapple juice orally, and suppressing pre-existing bowel fluid contents, with variable TI in three adult and one child volunteer.

**Results:**

Pineapple juice (PJ) had both the shortest T_1_ (243 ms) and shortest T_2_ (48 ms) of the fruit juices tested. Optimal signal differentiation between pre-existing bowel contents and oral PJ administration was obtained with TIs of between 900 and 1100 ms.

**Conclusion:**

The use of an inversion recovery preparation allowed long T_1_ pre-existing bowel contents to be suppressed whilst the short T_1_ of fruit juice acts as a positive contrast medium. Pineapple juice could be used as oral contrast agent for neonatal gastrointestinal magnetic resonance imaging.

## Background

Congenital abnormalities of gut rotation in neonates and infants are usually investigated by the upper gastrointestinal contrast study (UGI), using traditional X-ray fluoroscopy techniques. Malrotation is typically excluded on the basis of confidently identifying a normally positioned duodeno-jejunal (DJ) flexure, usually on the first pass of radio-opaque oral contrast medium through the duodenum at UGI [1]. However, these procedures require skilled interpretation, carry a significant radiation burden to the child, and may not always be diagnostic [2]. Optimal imaging of the neonatal GI tract is currently under review, with ongoing debate in the literature as to whether ultrasound or cross sectional imaging (such as CT) could replace the UGI series [3,4], although both have limitations, including ionising radiation doses in CT higher than that of UGI, and a significant false negative rate just as with UGI.

Recent advances in magnetic resonance imaging (MRI), particularly the development of interactive multi-contrast pulse sequences, may now provide an alternative imaging technique, thus avoiding ionising radiation. Interactive MRI allows real-time control over image plane location as well as a number of image acquisition parameters, including the development of an IR based Single Shot Fast Spin Echo (IR-SSFSE) sequence [5,6]. This could be useful in the setting of possible gut malrotation, just as interactive MRI has been used to visualise vesicoureteric reflux in the neonatal urinary tract [7]. However, in order to closely replicate the UGI series using MRI, an easily visualised contrast medium is needed.

The ideal medium should be easily tolerated, provide high positive signal enhancement, and help to distinguish the DJ flexure from pre-existing bowel contents in adjacent small bowel (likely to be a combination of milk or water-based material in neonates). There are several practical limitations in neonatal gut MRI, particularly regarding the choice of oral contrast agent. This is largely determined by their signal properties, the imaging modality used, the degree of gut distension required, and therefore the minimum volume of contrast agent required. Traditionally, barium sulphate is used in conventional radiographic gastrointestinal studies, but where there is a risk of spillage into the respiratory tract or perforation (particularly in paediatric studies) it has been replaced by non-ionic, low osmolality compounds (e.g. Iopamidol; Gastromiro, Bracco, High Wycombe, UK). High density barium has been used as a negative oral contrast agent for MR imaging of the gut [8], but typically requires high volumes of contrast medium to be ingested, which is not practical for neonates and small children.

Administering water (high contrast on T_2_w images) may not allow confident identification of the DJ flexure separately from the pre-existing fluid in adjacent jejunal loops, a key diagnostic factor for the UGI series. Currently, there are no gadolinium-based contrast agents licensed for oral use in children, but several commercially available (i.e. commodity) fruit juices have been used as both positive and negative oral contrast agents. For example, the paramagnetic properties of fruit juices such as concentrated pineapple or blueberry juice have been used to suppress the signal from bowel fluid in paediatric magnetic resonance cholangio-pancreatography (MRCP) studies which employ heavily T_2_ -weighted imaging [9,10]. As well as their T_2_ effects, the T_1_ shortening properties of these juices may also make them suitable positive contrast agents. However, they cannot be administered in high concentrations to neonates, due to the inability of the immature gut to deal with complex carbohydrate loads (of greater than 10%; [11]).

Administering an oral contrast medium with unknown positive signal properties, to an infant with unknown signal properties of small bowel content provides a wide range of variables. In this study, we first investigated the relaxation properties of several diluted fruit juices in vitro, to identify which could be used as oral contrast. We then describe our initial experience using an interactive MR pulse sequence, whereby real-time control over imaging parameters such as IR pulse timing could allow optimal contrast medium visualisation by suppressing pre-existing bowel fluid contents.

## Methods

We optimised the interactive IR-SSFSE sequence as part of an ethically approved study into optimising gut imaging (Cambridgeshire 3 Research Ethics Committee formal approval 08/H0306/7). Three adult volunteers and one child participated, who were nil-by-mouth for 6 hours and 3 hours respectively prior to the MR examination. Full informed written consent was obtained from each participant and the child’s parent. No sedation or anaesthesia was used. Ethical approval was not required for in-vitro studies.

### Contrast agent testing

**Figure 1:**
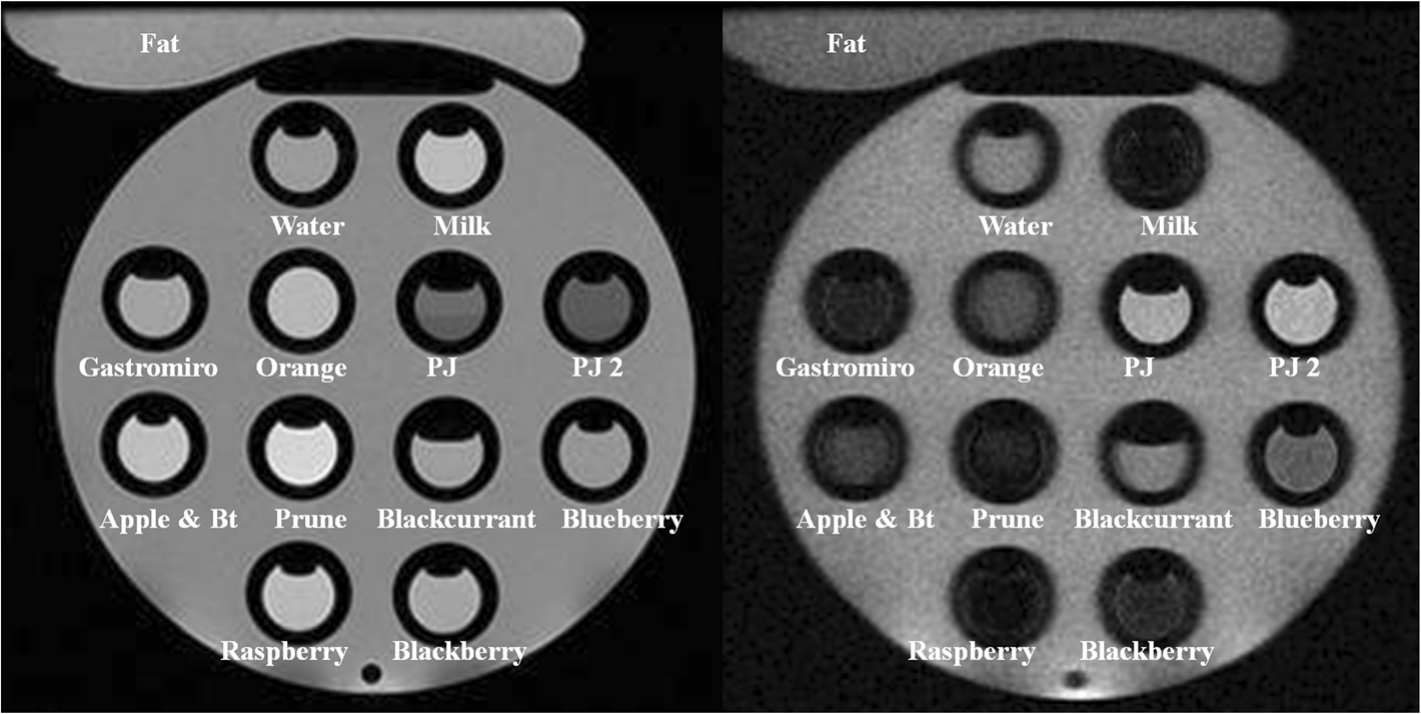
**12 different contrast media assessed in-vitro.** Pineapple juice (PJ and PJ2) have the lowest signal on T2w imaging (left : PDw SSFSE; TR 2500), but highest signal recovery on T1w imaging (right: IR-FSPGR with TI = 640 ms).

12 different contrast media were prepared, as follows: Tap water, standard infant formula milk (SMA Gold, SMA nutrition, Berkshire, UK), Iopamidol (Gastromiro, Bracco, High Wycombe, UK), and the following 9 commercially available (standard supermarket stock) fruit juices: orange juice, two examples of pineapple juice (one from juice; PJ, and one reconstituted from concentrate, PJ2), apple & beetroot juice, prune juice, blackcurrant juice, blueberry juice, raspberry juice, and blackberry juice. Three of these products (PJ2, prune, and blueberry juice) required further dilution to achieve a carbohydrate concentration of <10%, all other media were imaged undiluted (see Table 1). Estimated nutritional and paramagnetic agent contents of each juice are given (Table 2).Each proposed contrast agent was placed within a multi-compartment phantom positioned inside an 8 channel brain array coil, and images acquired on a standard 1.5 T whole-body MR system (Signa HDx; GE Healthcare, Waukesha, WI; Figure 1).

**Table 1:** **Carbohydrate (COH) concentration, and measured T**_**1 **_**and T**_**2 **_**relaxation times of 12 different contrast media**

	**Solution**	**COH conc (per 100 ml)**	**T**_**1 **_**(ms)**	**T**_**2 **_**(ms)**
1	Water	0	2831	1950
2	Milk	7.3%	930	148
3	Iopamidol	0	810	165
4	Orange	9.0%	1796	440
5	PJ	10.0% (12.7%*)	243	48
6	PJ2	8.6%	258	53
7	Apple/beetroot	9.6%	1679	518
8	Prune	10.0% (16.8%*)	1245	324
9	Blackcurrant	1.0%	2803	1480
10	Blueberry	10.0% (10.5%*)	573	80
11	Raspberry	0.3%	1055	146
12	Blackberry	0	831	103

COH concentration was provided from manufacturers packaging, and not tested directly.

3 juices (*) had too high a COH concentration, and so were diluted down to 10% prior to testing: the original concentrations are given in parentheses.

**Table 2:** Contents of each juice tested as provided by manufacturer

	**Solution**	**Manufacturer**	**Description as purchased**	**Energy**	**Protein**	**COH**	**Fat**	**Iron**	**Manganese**	**Copper**
				**(kcal)**	**(g)**	**(g)**	**(g)**	**(mg)**	**(mg)**	**(mg)**
2	Milk	SMA Gold	SMA Gold	67	1.9	7.3	3.3	0.64	0.09	0.06
4	Orange	Sainsburys	Pure orange juice (conc)	47	0.5	9.0	trace	0.12	0.04	0.02
5	PJ	Sainsburys	Pure pineapple juice (conc)	54	0.1	12.7	0.0	0.29	0.92	0.11
6	PJ2	Marks & Spencers	99% pineapple juice	35	0.5	8.6	0.1	0.29	0.92	0.11
7	Apple/beetroot	Marks & Spencers	Pressed apple & beetroot	40	0.3	9.6	0.1	0.12	0.07	0.01
8	Prune	Tesco	Pure prune juice	75	0.7	16.8	0.1	1.18	0.15	0.07
9	Blackcurrant	Disney	Blackcurrant with sweetener	2	trace	1.0	trace	1.54	0.26	0.08
10	Blueberry	Sainsburys	Blueberry juice drink (conc)	44	0.1	10.5	trace	0.28	0.33	0.06
11	Raspberry	Sainsburys	Juice	30	0.4	0.3	0	0.35	0.33	0.04
12	Blackberry	Twinings tea	2% blackberry flavouring, 10% blackberry leaves	0	0	0	0	0.31	0.32	0.08

Water and Iopamidol have been excluded from the table as no nutritional information is provided. All values are given per 100 ml from concentrate. Estimated iron, manganese and copper content of standard juices were obtained from the USDA National Nutrient Database ( http://ndb.nal.usda.gov/).

The T_1_ values were measured using a 2D inversion recovery (IR) prepared fast radiofrequency (RF) spoiled gradient echo (FSPGR) sequence with variable inversion times (TI) at nine different TIs (40, 80, 160, 320, 640 ms, and 1.28, 2.56, 5.12, 10.24 sec). Other imaging parameters were: field of view (FOV) 25×25 cm, slice thickness 5 mm, matrix 256 × 256, TR 7.2, TE 3.3 ms.

The T_2_ values were measured using an 8-echo spin echo sequence. Eight different echo times (TE) were used, 30, 60, 90, 120, 150, 180, 210, 240 ms. Other imaging parameters were: FOV 25×25 cm, slice thickness 5 mm, matrix 256 × 256, TR 2000 ms.

We then measured the signal intensity of each contrast medium using the IR-SSFSE sequences at variable TI times (0–4000 ms), to simulate the in-vivo environment, and subtracted the signal intensity of milk to predict the possible optimal TI times in vivo.

### Interactive IR-SSFSE

**Figure 2:**
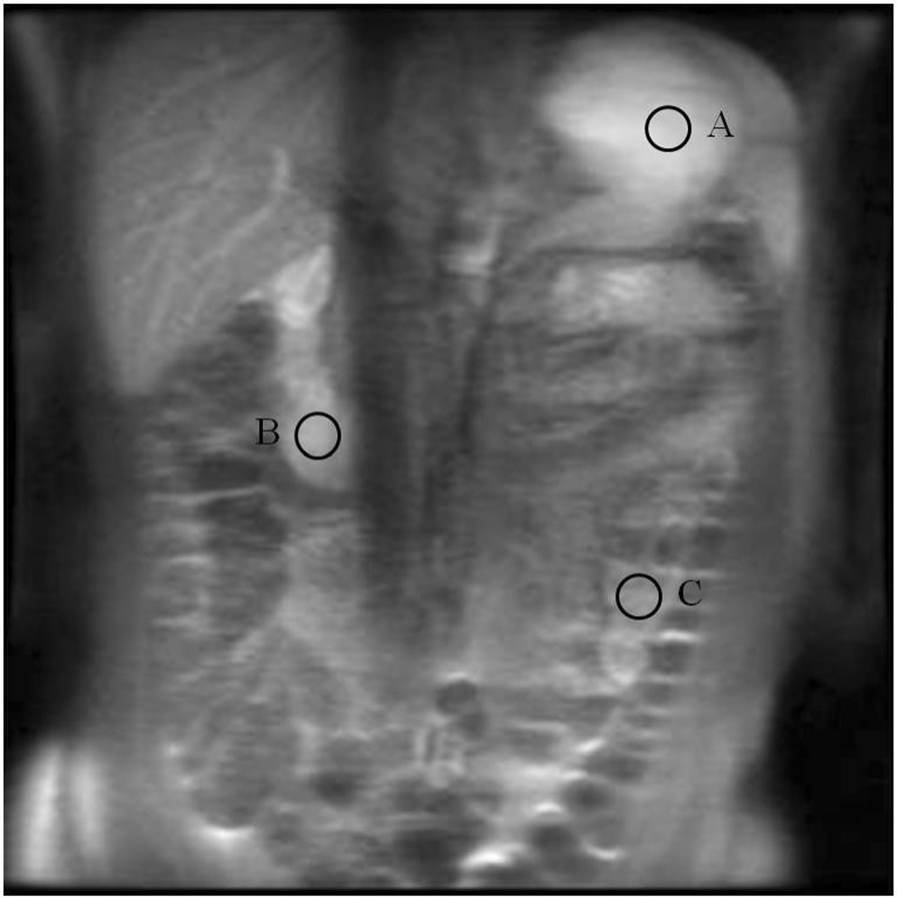
**Coronal IR-SSFSE images through the abdomen in an adult following oral pineapple juice administration.** Signal intensity was measured in 3 regions of interest: the stomach **(A)**, proximal small bowel (duodenum; **B**) and mid-small bowel (jejunum; **C**).

Interactive MRI in-vivo was performed using an IR-SSFSE pulse sequence. This modified pulse sequence has been custom developed to operate within a proprietary interactive imaging interface (i/Drive Pro Plus; GE Healthcare) which allows interactive control over many imaging parameters such as image plane location, phase encode ordering, and the use of a 180° inversion pulse that can be interactively toggled on and off prior to the SSFSE readout. The inversion time (TI) between the 180° inversion pulse and the SSFSE readout could also be interactively controlled during imaging, by inputting different IR times in ms. Initial imaging parameters for the SSFSE sequence were: FOV 25×25 cm, matrix 256×256, TR 2500 ms, TE 50.1 ms, single slice thickness of 10 mm. No motion suppression or breathing suppression technique was used.In volunteer imaging, initial localiser images and non-interactive axial and coronal FIESTA and SSFSE sequences were obtained in a supine position to outline upper gastrointestinal anatomy and localise pre-existing small bowel contents. Coronal interactive IR-SSFSE was then used during free breathing to vary the TI at 100 ms intervals (between 0 and 2000 ms), and signal intensities were measured in 3 regions of interest (ROIs): stomach, proximal small bowel (duodenum) and mid-small bowel (jejunum; Figure 2), in order to characterise the signal properties of pre-existing bowel contents.

We then repeated the study with increasing TIs within 10 minutes of the oral administration of 200 ml pineapple juice to three adult volunteers, and after 20 ml pineapple juice administration to a single neonatal infant (2 week old 3 kg male infant). We did not use any form of respiratory or cardiac gating.

## Results

### In vitro testing

**Figure 3:**
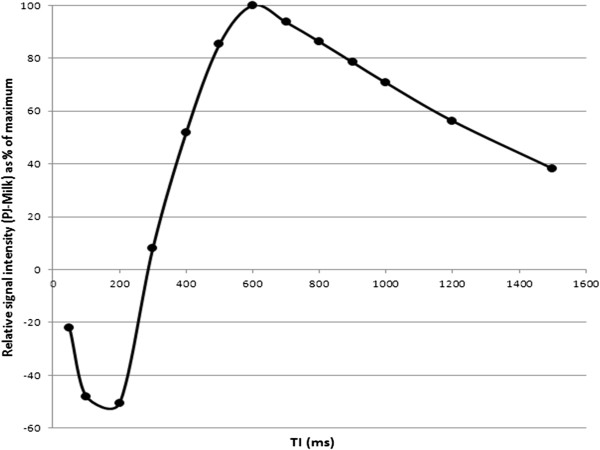
**By subtracting the signal intensity of PJ and milk, we were able to plot the relative differences in signal (plotted as a % of maximum) between the two contrast media at different TIs.** Maximal contrast differences are seen with TI between 500 – 900 ms.

Figure 1 demonstrates the phantom used for in-vitro testing. The T_1_ and T_2_ relaxation times for the contrast media tested are reported in Table 1: pineapple juice from juice (PJ) was found to have both the shortest T_1_ (243 ms) and T_2_ (48 ms) relaxation times.Assuming that milk was likely to be the main constituent of pre-existing bowel contents in pre-weaned infants, a representative graph of the relative difference in signal intensity between PJ and formula milk is shown in Figure 3, demonstrating that maximal differences are seen in-vitro with TIs between 500 – 900 ms.

### In vivo strategy

**Figure 4:**
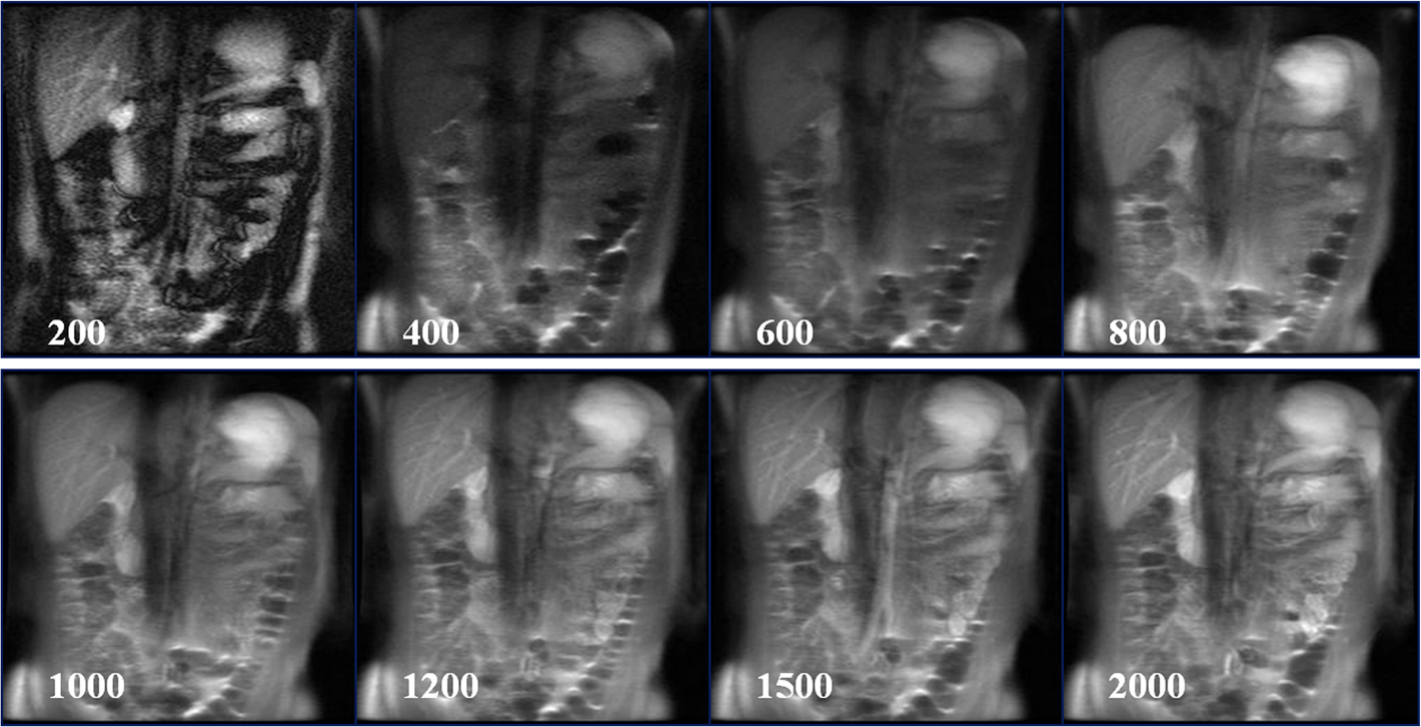
**Coronal IR-SSFSE images (TI = 1200 ms) through the abdomen in an adult following oral pineapple juice administration.** Examples of different TI times (200 – 2000 ms) are given. Relative signal intensity from this study is given in Figure 5.

**Figure 5:**
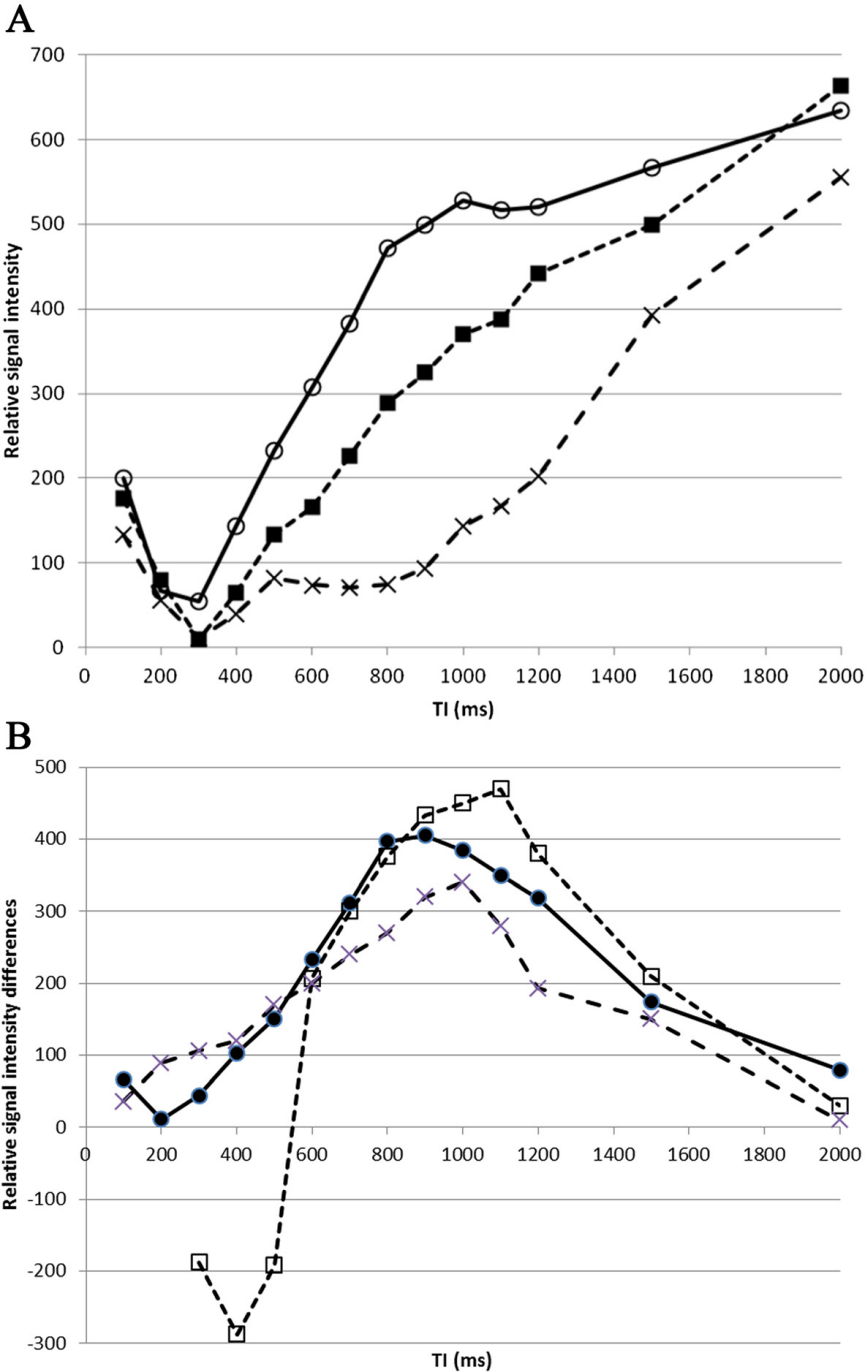
**A. Relative signal intensity in three gut regions (A, B and C in Figure**2**) following oral pineapple juice administration.** ROIs were A: stomach (open circles, solid line), B : proximal small bowel (filled squares, dashed line), and C: more distal small bowel (cross, dotted line). **B**. Relative signal intensity *differences* (as a % of maximum) between stomach and distal small bowel (A – C; filled circles, solid line), and between proximal and more distal small bowel (B – C; open squares, dashed line).

**Figure 6:**
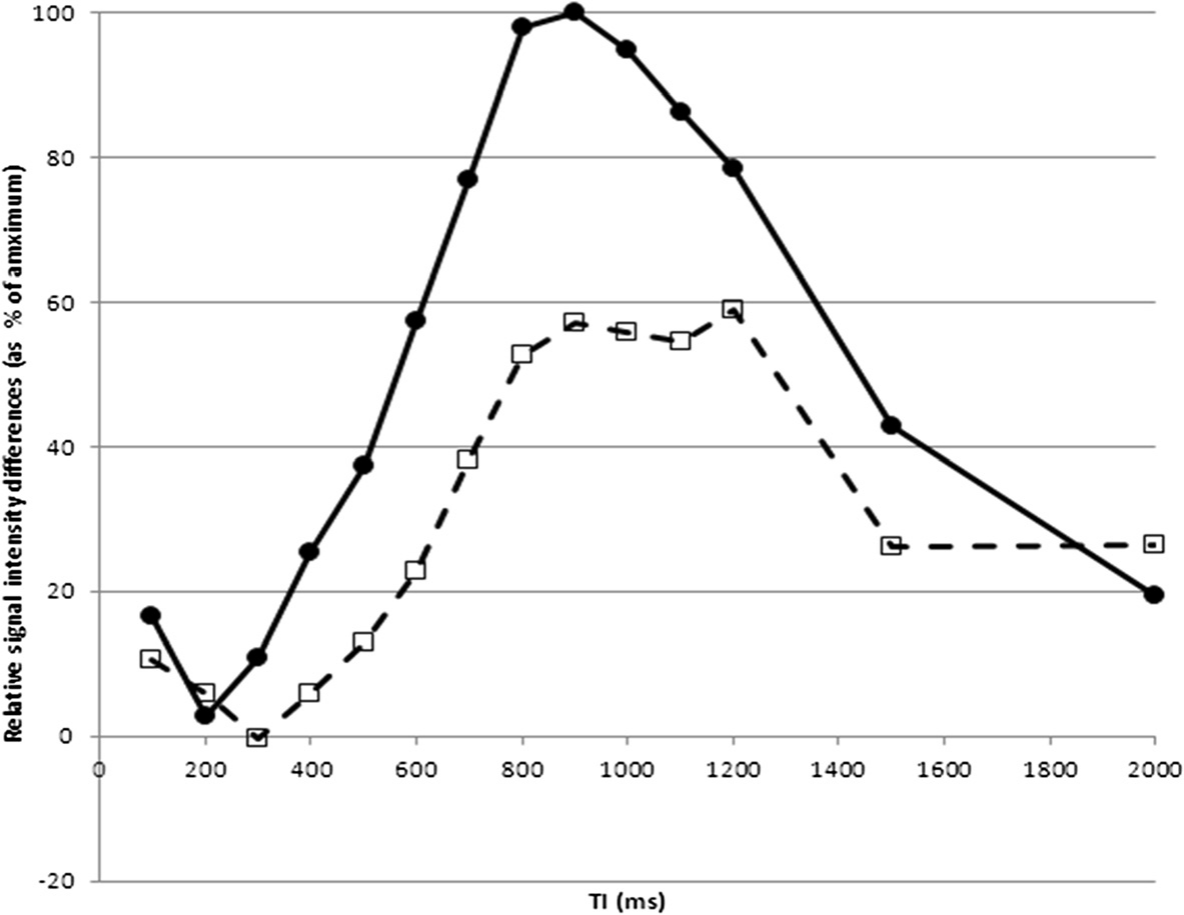
**Relative signal intensity *differences *between stomach and proximal small bowel, with maximum differences observed at TIs of 1000 ms, 1100 ms, and 900 ms in three individual adult volunteers.** The shape of each graph was similar, with signal brighter in small bowel than stomach in one individual, which may suggest rapid transit time. The consistent maximum differences at TI of 900 – 1100 ms are roughly in keeping with the in-vitro results.

**Figure 7:**
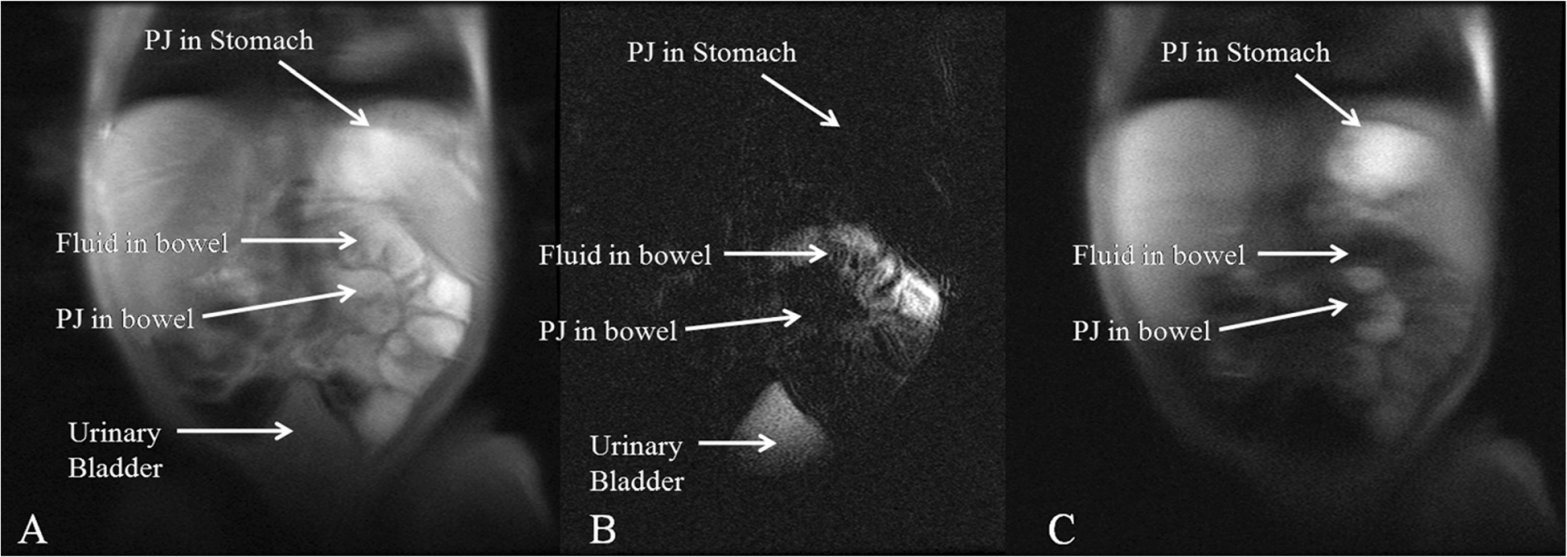
**Coronal IR-SSFSE of neonatal GI tract.** A 2 week old 3 kg male infant underwent MRI following PJ administration. **A**. Conventional SSFSE (TR 2500 ms) imaging. It is not possible to differentiate between PJ in the stomach and proximal small bowel from fluid already in the small bowel on conventional T2w imaging. **B**. T_2_w hydrographic SSFSE image (TR of 4000 ms) demonstrating location of pre-existing bowel fluid. The short T_2_ PJ in the stomach and proximal bowel is low signal. **C**. IR-SSFSE PDw (TI = 1000 ms) that nulls the signal from pre-existing bowel fluid, and demonstrates PJ as high signal in stomach and proximal bowel.

We subsequently tested pineapple juice as an in vivo contrast agent using the IR-SSFSE sequence: we measured signal intensity in three different ROIs (Figures 2), with increasing T1 at 100 ms intervals (0 – 2000 ms; Figure 4) before and after oral PJ administration. By subtracting the signal intensity of distal (jejunal) small bowel contents, we plotted differences in signal intensity (Figure 5) and showed that maximum differences between stomach and jejunum contents were observed at TIs of 1000 ms, 1100 ms, and 900 ms in the three adult volunteers respectively (Figure 6). We were unable to obtain good images of every TI in the neonate following 20 ml PJ administration due to patient motion, but maximal signal differences were seen at 1000 ms (Figure 7).

## Discussion

This study demonstrates that pineapple juice could be used as a positive contrast agent for the proximal neonatal gut. By tailoring an IR-SSFSE sequence to null the signal from existing bowel fluid and thereby optimise the signal from PJ, we have developed a strategy for bowel visualisation using MRI which could be used in a similar fashion to the traditional UGI series in children. The true diagnostic performance of an interactive MRI strategy under these conditions remains to be evaluated.

IR-SSFSE sequences have previously been used for MR imaging of the abdomen [12], but not with interactive control. Our strategy used the IR-SSFSE sequence to effectively suppress bowel fluid signal where its properties were unknown, and to optimally tune the IR timing to provide optimal signal from a contrast agent with known signal properties. Several vendors may offer an IR-prepared sequence, such as T1-scout series or modified look-locker inversion recovery (MOLLI) sequences, where changes in inversion recovery time can be used to null myocardium during delayed enhanced imaging, for instance. However they do not allow for on-the-fly adjustment of the TI, which is necessary for rapid optimisation in a paediatric setting. Our strategy helps to tailor the examination to the individual, and has the potential to reduce overall examination time, although this remains to be evaluated in clinical practice.

One of the advantages of using the T_1_ shortening properties of fruit juices are that smaller volume can be used as gut distension is not a requirement. Here, we were able to administer 200 ml to an adult, less than some studies using blueberry juice in adults (400–600 ml) [9,13,14]. Using only 20 ml in a neonate is comparable to that used in an UGI and is less than normal meal intakes. The majority of other substances used for MR gut imaging are typically combined with gut-distending agents such as mannitol, known to give gastrointestinal side effects such as flatulence and bloating, but still widely used for small bowel MR studies in older children and adults.

Despite differences in commercial products and dilutions, our measurements closely mirror those found in other studies, particularly regarding the short T_1_ and T_2_ of PJ [15]. We included formula milk in our evaluation and obtained similar values to the range previously measured (640–1750 ms) [16]. Several other fruit juices have been proposed as good gut contrast agents, but have only been evaluated in adults, including concentrated blueberry juice and blackberry juice [13,17,18].

The main difference between our phantom study and some previous experiments is likely to relate to the dilutions used; several previous studies measured the signal characteristics of concentrated fruit juices, or indeed fruit pulp itself [17]. Concentrated fruit juices can stimulate gut transit in non-weaned infants, causing diarrhoea and nappy irritation, which can be both uncomfortable and distressing for the child and care-giver, which is why we used diluted products. It is widely reported that the signal suppression effect is due to T_2_ relaxation caused by their inherently high iron (blackberry) or manganese (pineapple, beet juice, green tea) content, although the exact contents of different commercial fruit juices vary widely, and the precise paramagnetic component (iron, copper or manganese) that gives the relaxation shortening property is debatable. We estimated the paramagnetic agent contents of each of our fruit juices (Table 2) and hypothesise that it may be the manganese content of pineapple juice which gave favourable properties in this study.

Several other oral agents have been developed and have been comprehensively reviewed elsewhere [19]. These can be grouped into predominantly *positive* gastrointestinal MR imaging contrast media, typically gadolinium-based compounds e.g. Magnevist Enteral® (Schering AG, Berlin, Germany) or Gadolinium zeolite (Gadolite®; Pharmacyclics, Sunnyvale, CA, now GlaxoSmithKline Ltd, Brentford, UK), or *negative* gastrointestinal MR imaging contrast agents, particularly iron-containing solutions such as Perflubron® (Imagent GI®, Alliance Pharma), or Abdoscan® (Nycomed-Amersham, Oslo, Norway). Most of these are not commercially available, and are not licensed for children. Carbon dioxide gas has also been used as a negative contrast agent for proximal gut MR imaging and early studies demonstrated acceptable visualisation of the duodenum but were limited in the jejunum and small bowel [20], but this remains to be investigated in neonates.

### Limitations of this study

One limitation of this feasibility study could be the variable content of commercially available fruit juice. We did not determine the exact constituents of the different fruit juices used in this study, but relied on the manufacturers details provided on the packaging and estimated provided by the USDA National Nutrient Database ( http://ndb.nal.usda.gov). Since the concentrations of fruit pulp and therefore paramagnetic properties of diluted commercial fruit juices may vary, the ability to interactively optimise the IR timing becomes even more important as a way to compensate for these variations.

We acknowledge that we did not include any patients in this volunteer feasibility study, and have tested only a limited number of volunteers. The full diagnostic potential of this technique now needs to be evaluated in a larger patient population.

## Conclusion

In conclusion, this work indicates that pineapple juice is the most promising natural commercially available short T_1_ contrast medium suitable for neonatal GI tract imaging using an interactively adjusted inversion-recovery SSFSE strategy. This allows for pre-existing longer T_1_, bowel contents to be suppressed, whilst the short T_1_ of the PJ acts as a positive contrast medium to delineate the gut. This approach allows individual tailoring of the examination, to give optimal discrimination of an orally administered contrast agent from the background signal of gut contents. Further work is required using this strategy in a larger cohort of neonates and infants to evaluate both technical and diagnostic performance.

## Competing interests

The authors declare that they have no competing interests, financial or non-financial

## Authors’ contributions

OA, MJG, IJ and ADE carried out the in-vitro studies, and OA, MJG, IJ and PS carried out the in-vivo interactive MRI studies. All authors participated in the design of the study and helped to draft the manuscript. All authors read and approved the final manuscript.

## Pre-publication history

The pre-publication history for this paper can be accessed here:

http://www.biomedcentral.com/1471-2342/14/33/prepub
